# Supplementary material for: Identifying Protein Features and Pathways Responsible for Toxicity Using Machine Learning and Tox21: Implications for Predictive Toxicology
Source: Molecules. 2022 May 8;27(9):3021. doi: 10.3390/molecules27093021 (PMC9099959; doi:10.3390/molecules27093021)
Supplement: Supplementary file 1 [file molecules-27-03021-s001.zip › molecules-1637614-supplementary.pdf]

Supplementary Materials: Identifying Protein Features and Pathways Responsible for Toxicity Using Machine Learning and Tox21: Implications for Predictive Toxicology

Lama Moukheiber [1] , William Mangione [2] , Mira Moukheiber [2], Saeed Maleki [3], Zackary Falls [2], Mingchen Gao [1,*] and Ram Samudrala [2,*]

Table S1. Protein identifiers and names associated with the NR-AhR toxicity pathway enrichment analysis.

Gene identifier	UniProt ID	Protein name
BRCA1	P38398	Breast cancer type 1 susceptibility protein
CNTN1	Q12860	Contactin-1
GINS2	Q9Y248	DNA replication complex GINS protein PSF2
GLB1	P16278	Beta-galactosidase
GP1BB	P13224	Platelet glycoprotein Ib beta chain
LGR5	O75473	Leucine-rich repeat-containing G-protein coupled receptor 5
LRP6	O75581	Low-density lipoprotein receptor-related protein 6
NANP	Q8TBE9	N-acylneuraminate-9-phosphatase
NCAM1	P13591	Neural cell adhesion molecule 1
NRCAM	Q92823	Neuronal cell adhesion molecule
NRP1	O14786	Neuropilin-1
NSD2	O96028	Histone-lysine N-methyltransferase NSD2
PCGF5	Q86SE9	Polycomb group RING finger protein 5
PIAS3	Q9Y6X2	E3 SUMO-protein ligase PIAS3
PROCR	Q9UNN8	Endothelial protein C receptor
RBX1	P62877	E3 ubiquitin-protein ligase RBX1
RNF125	Q96EQ8	E3 ubiquitin-protein ligase RNF125
RNF2	Q99496	E3 ubiquitin-protein ligase RING2
RNF8	O76064	E3 ubiquitin-protein ligase RNF8
RPA1	P27694	Replication protein A 70 kDa DNA-binding subunit
TEAD1	P28347	Transcriptional enhancer factor TEF-1
TEAD4	Q15561	Transcriptional enhancer factor TEF-3
TRAF2	Q12933	TNF receptor-associated factor 2
TRAF6	Q9Y4K3	TNF receptor-associated factor 6
UBE2N	P61088	Ubiquitin-conjugating enzyme E2 N
UHRF2	Q96PU4	E3 ubiquitin-protein ligase UHRF2
